# Partial Kluver-Bucy syndrome as a delayed manifestation of head injury

**DOI:** 10.4103/0972-6748.62272

**Published:** 2009

**Authors:** P. S. Bhat, P. K. Pardal, R. C. Das

**Affiliations:** Department of Psychiatry, AFMC, Pune, India; 1Shri RamaMurthy Medical College, Bareilley (UP), India; 2Command Hospital (SC), Pune, India

**Keywords:** Kluver-Bucy syndrome, Traumatic brain injury, Neurobehavioural syndrome

## Abstract

After traumatic brain injury (TBI), the most disabling problems are generally related to neuropsychiatric sequelae, including personality change and cognitive impairment, rather than neurophysical sequelae. Kluver-Bucy syndrome (KBS) is a rare neurobehavioral condition, first described in 1937 as an experimental neurobehavioral syndrome in monkeys with bitemporal brain lesions. The syndrome in man was subsequently observed to be transient or permanent in a variety of neurodegenerative disorders and after traumatic, nontraumatic and infectious brain injury. However, partial KBS may occur in the absence of the classic bilateral temporal lesion, though rare. Pharmacological treatment of post-TBI neuropsychiatric sequelae consists of symptomatic, functional and hypothetical approaches. Specific pharmacological treatment consists of antipsychotics, anti-kindling anticonvulsants or a combination thereof. A case of partial KBS presenting as delayed manifestation of traumatic brain injury that improved with carbamazapine and antipsychotics is presented.

Traumatic brain injury (TBI) is a major public health issue. In the long term, after traumatic brain injury, the most disabling problems are generally related to neuropsychiatric sequelae, including personality change and cognitive impairment, rather than neurophysical sequelae (Fleminger, 2008). In some individuals, the TBI may cause decades-lasting vulnerability to psychiatric illnesses (Koponen *et al*., 2002). Recent evidence from clinical studies and animal models of brain injury suggests that neuronal and glial loss might progress after the initial insult in selectively vulnerable regions of the brain, such as the hippocampus (Jorge *et al*., 2007).

Kluver-Bucy syndrome (KBS) is a rare neurobehavioral condition, first described in 1937 as an experimental neuro behaviour syndrome in monkeys with bitemporal brain lesions. The syndrome in man was subsequently observed to be transient or permanent in a variety of neurodegenerative disorders and after traumatic, nontraumatic and infectious brain injury (Gaul *et al*., 2007). However, partial KBS may occur in the absence of the classic bilateral temporal lesion, though rare (Carroll *et al*., 2001). We present a case of partial KBS presenting as delayed manifestation of traumatic brain injury.

## CASE REPORT

A 30-year-old male soldier presented with an acute-onset gross behavioral abnormality of 1-week duration. He was noted to be irritable, showing fluctuating level of aggression, and lacking social etiquettes. He was touching his faeces, licking it and applying it over the clothes. After initial supportive measures, he was referred for psychiatric evaluation.

Physical examination was within normal limits (WNL). Mental status examination showed an ill-kempt unshaven individual; flattened affect; inability to recognize colleagues; intact sensorium; variable psychomotor activity, from complete withdrawal state to unprovoked aggression; no evidence of delusions or hallucinations; touching and rolling the objects in vicinity; irrelevant speech; lack of insight; and deranged biodrives. In the ward, he passed urine into a mug and washed his face. He was found to be putting inanimate articles into the mouth, and he even bit the escorts. He was noted to be fondling the genitals in public. No relevant past or family history could be obtained initially. Investigations including hemograms, urinalysis, blood sugar, blood urea were WNL. CT head showed “mild atrophy of right anterior temporal gyrus.” EEG was WNL. MRI confirmed CT findings and ruled out any other lesions.

Behavioral control was achieved with neuroleptics quickly. The patient then gave past history of having met with a road traffic accident about a year back on a home visit while on leave and sustaining a closed head Injury. CT head then had revealed hemorrhagic contusions in the right frontoparietal region. He had been managed conservatively and he recovered. There was no other significant past or family history.

He was diagnosed as a case of organic brain syndrome as a consequence of head injury. In the background of posttraumatic right-sided anterior temporal gyrus atrophy, increased oral activity, hypersexuality, features of hypermetamorphosis, loss of recognition of people, a diagnosis of partial Kluver-Bucy syndrome was entertained.

He was treated with anti-kindling mood stabilizer carbamazapine and other supportive measures. He responded quickly and became asymptomatic within 3 weeks. Subsequent follow-up after convalescence leave did not reveal any residual neurobehavioral abnormality after gradual tapering off of the medication.

## DISCUSSION

Kluver and Bucy described a neurobehavioral syndrome following bitemporal lobectomy in monkeys in 1937, which was later named after them. Extrapolating any syndrome from monkey to man is fraught with controversy, but Aichner in 1984 after studying 53 patients with KBS described the phenomenology in detail (Aichner, 1984). It includes the following symptoms: (a) Increased oral activity — a strong tendency to examine all objects orally, putting objects in mouth, licking, biting, chewing and touching with lips; (b) hypersexuality — hetero, homo or autosexual; (c) hypermetamorphosis — to touch everything in sight, to attend to every visual stimulus with grasping, hugging movements; (d) memory disorders; (e) placidity — flattened affect; (f) loss of recognition of people; and (g) bulimia. The KBS in man shows little etiologic specificity and has been seen with posttraumatic encephalopathy, encephalitis, anoxia and subarachnoid hemorrhage. Since the lesions in man are not as extensive and not as consistently localized as in animal experiments, it is not surprising that all abnormal behavioral deficits following bitemporal lobectomy have seldom been exactly reproduced in man. Hence the full syndrome is rarely seen; and to indicate a partial KBS in man, combinations of 3 or more different elements must be present.

Following a TBI, a chronically progressive degenerative process may be initiated (Smith *et al*., 1997). It has been reported that even a single episode of closed head trauma induces long-lasting alterations in the hippocampus (Santhakumar *et al*., 2001). KBS following unilateral temporal lobe injury has been reported by many (Anson *et al*., 1993; Salim *et al*., 2002; Ghika-Schmid *et al*., 1995). Though the exact mechanism has not been established, a transient functional bilateral temporal lobectomy has been postulated (Anson *et al*., 1993).

Pharmacological treatment of post-TBI neuropsychiatric sequelae consists of symptomatic, functional and hypothetical approaches. Specific pharmacological treatment consists of antipsychotics, anti-kindling anticonvulsants or a combination thereof (Ahmed *et al*., 1998). Carbamazapine has been used with good success by many (Carroll *et al*., 2001; Stewart, 1985; Góscínski *et al*., 1997). There is a need to follow up cases of head injury for a longer duration.
